# Midgut microbiota diversity of potato tuber moth associated with potato tissue consumed

**DOI:** 10.1186/s12866-020-01740-8

**Published:** 2020-03-11

**Authors:** Yaqiang Zheng, Guanli Xiao, Wenwu Zhou, Yulin Gao, Zhengyue Li, Guangzu Du, Bin Chen

**Affiliations:** 1 Key Laboratory of Agro-biodiversity and Pest Management of China’s Ministry of Education, College of Plant Protection, Yunnan Agricultural University, Kunming, 650201 China; 2 College of Agriculture & Biology Technology, Yunnan Agricultural University, Kunming, 650201 China; 3 College of Agriculture & Biology Technology, Zhejiang University, Hangzhou, 310058 China; 4 State Key Laboratory for Biology of Plant Diseases and Insect Pests, Institute of Plant Protection, Chinese Academy of Agricultural Sciences, Beijing, 100081 China

**Keywords:** Potato tuber moth, *Phthorimaea operculella* (Zeller), Potato, Insect, Midgut bacteria, Endophytic bacteria

## Abstract

**Background:**

The potato tuber moth (PTM), *Phthorimaea operculella* (Zeller), is a worldwide pest that feeds on both the leaves and tubers of potato plants. PTM larvae can digest leaves, or tubers, resulting in serious damage to potato plants in the field and potato tubers in storage. To understand how midgut bacterial diversity is influenced by the consumption of these two tissue types, the symbiotic bacteria in the potato-feeding PTM midgut and the endophytic bacteria of potato tissues were analyzed.

**Results:**

At the genus level, the bacterial community composition in the PTM midgut was influenced by the tissues consumed, owing to their different nutrient contents. *Escherichia_Shigella* and *Enterobacter* were the most dominant genera in the midgut of leaf-feeding and tuber-feeding PTMs, respectively. Interestingly, even though only present in low abundance in leaves and tubers, *Escherichia*_*Shigella* were dominantly distributed only in the midgut of leaf-feeding PTMs, indicating that specific accumulation of these genera have occurred by feeding on leaves. Moreover, *Enterobacter,* the most dominant genus in the midgut of tuber-feeding PTMs, was undetectable in all potato tissues, indicating it is gut-specific origin and tuber feeding-specific accumulation. Both *Escherichia_Shigella* and *Enterobacter* abundances were positively correlated with the dominant contents of potato leaves and tubers, respectively.

**Conclusions:**

Enrichment of specific PTM midgut bacterial communities was related to different nutrient levels in different tissues consumed by the insect, which in turn influenced host utilization. We provide evidence that a portion of the intestinal microbes of PTMs may be derived from potato endophytic bacteria and improve the understanding of the relationship between potato endophytic bacteria and the gut microbiota of PTMs, which may offer support for integrated management of this worldwide pest.

## Background

The potato tuber moth (PTM), *Phthorimaea operculella* (Zeller), is the most destructive and ubiquitous pest of solanaceous crops and is especially devastating to potatoes (*Solanum tuberosum*) [1]. PTM larvae can mine leaves, stems, petioles, or tubers, and cause serious damage to potato plants in the field and potato tubers in storage. As a result, tuber quality is reduced and the risk of pathogen infection is increased [2–4]. Previous findings revealed no significant difference in the life history and fecundity of PTMs living on potato leaves and tubers [2, 3], although a significant difference was found in the nutrient contents of potato leaves and tubers [5, 6]. It remains unclear how PTMs adapt to these two different food sources.

In recent years, many studies have shown that intestinal microbes play important roles in insects [7]. These microbes can degrade complex plant polysaccharides [8, 9] detoxify plant toxins [10, 11], synthesize nutrients required by insects [12, 13], mediate insect resistance to insecticides [14–16], affect mating behaviors [17], and promote host weight gain [18]. However it remains unclear which factors affect the gut microbiota of PTMs. Sevim et al. examined PTM intestinal microbes using culture-based methods, and 8 strains representing 7 genera were cultured [19]. However, little information regarding the function of the gut microbiota of PTMs is presently available.

Some endophytic bacteria from plant tissues might be the same as those present in the insect gut [20], and can establish microbial communities in plant-feeding insect guts after transference from a plant [21]. Some studies have shown that many bacterial endophytes colonize the inner tissues of potato organs [22–24] and can promote growth and enhanced resistance of potato plants to pathogenic bacteria and fungi [25–27]. However, little is known regarding the interaction of bacterial endophytes from potato plants and insects that feed on potatoes. In addition, the PTM is oligophagous and thrives on solanaceous crops, making them especially devastating to potato harvests. Consequently, studying the interaction between gut microorganisms and PTM endophytes is particularly important.

In this study, the development of PTMs on tubers and leaves of two potato cultivars (Hezuo-88 and Lishu-6) was observed, and the nutrients present in potato leaves and tubers were measured. The structure of bacterial communities in the midgut of 4th-instar PTMs feeding on potato tubers and leaves, and endophytic bacteria of potato tubers and leaves were examined using Illumina high-throughput sequencing of the 16S rRNA gene. Moreover, the relationship between nutrient contents and the bacterial community composition was analyzed to determine the role of intestinal microbes in the adaptation of the PTM diet to leaves and tubers. The endophytic bacteria of different potato organs and intestinal midgut microbes of potato-feeding PTMs were compared.

## Results

### PTM feeding on leaves and tubers of different potato cultivars

**Fig. 1:**
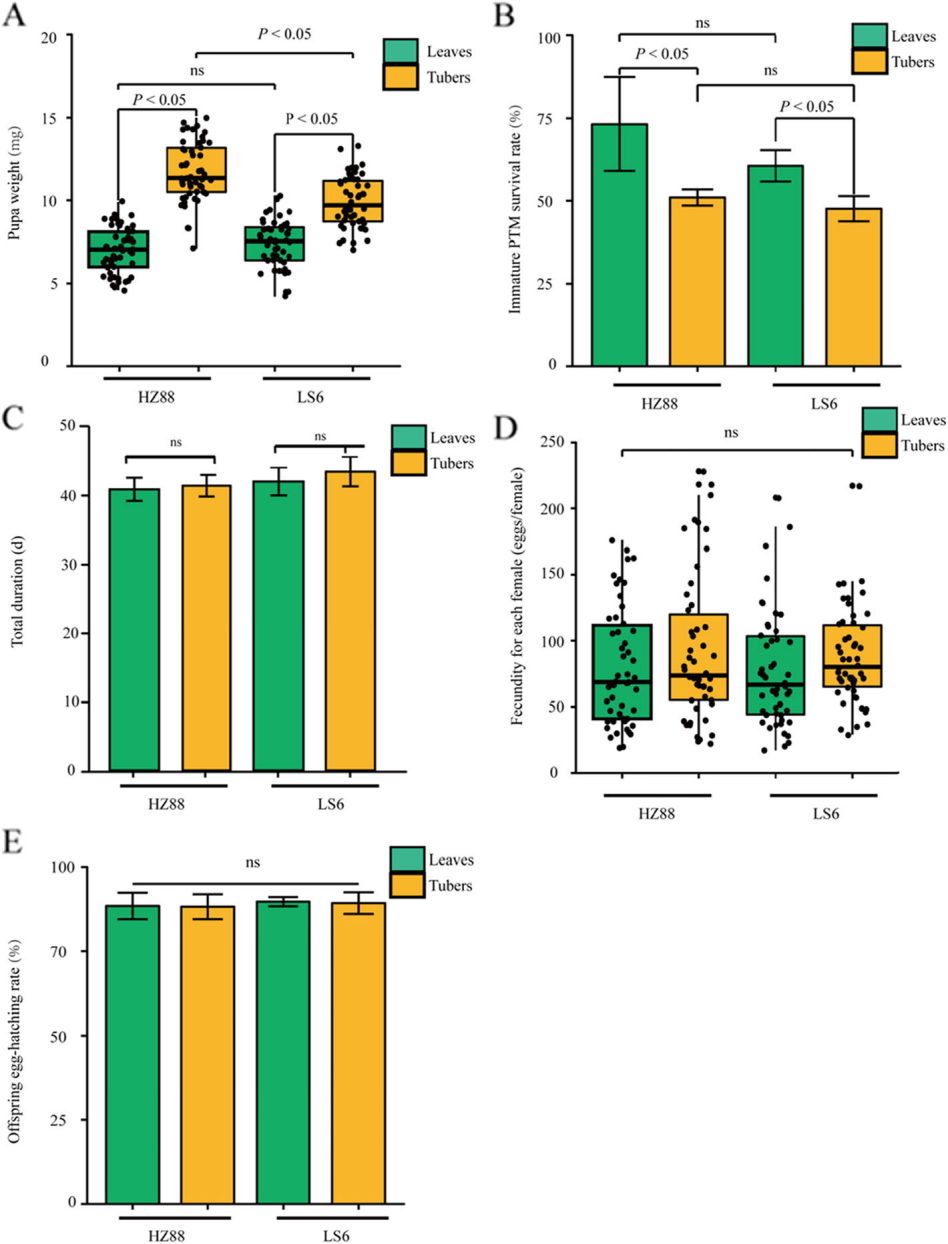
Performance of PTMs living on tubers and leaves of different potato cultivars. **a** Weights of PTM pupa living on the leaves and tubers of two different potato varieties. Each point denotes a biological replicate measurement of PTM pupa weight. **b** The survival rate of PTMs living on leaves and tubers of two different potato varieties. **c** Total duration of PTM growth on leaves and tubers of two different potato varieties. **d** The total fecundity per female PTM living on leaves and tubers of two different potato varieties. Each point denotes a biological replicate measurement of total fecundity per female. **e** The offspring egg-hatch rates of PTMs living on leaves and tubers of two different potato varieties. HZ88 and LS6 refer to the HZ-88 and LS-6 potato varieties, respectively. Statistical differences were assessed using Student’s *T*-test, with *P* < 0.05 considered a significant difference and labeled “*P* < 0.05”, with *P* > 0.05 considered not significant difference and labeled “ns”

The development period, pupa weight, survival rate, fecundity, and offspring egg-hatching rate were analyzed to understand the influence of tissue-specific feeding on the performance of PTMs (Fig. 1). The pupa weights of tuber-feeding PTMs were higher than those of leaf-feeding PTMs. The survival rate of leaf-feeding PTMs were higher than those of tuber-feeding PTMs. However, no significant differences were found during the development period, fecundity and offspring egg-hatching rates between the tuber- and leaf-feeding PTMs. Moreover, PTM larvae showed similar performances after feeding on the Hezuo-88 (HZ-88) and Lishu-6 (LS-6) potato cultivars.

### Basic statistics of the V3–V4 regions of 16S rRNA gene sequences

**Fig. 2:**
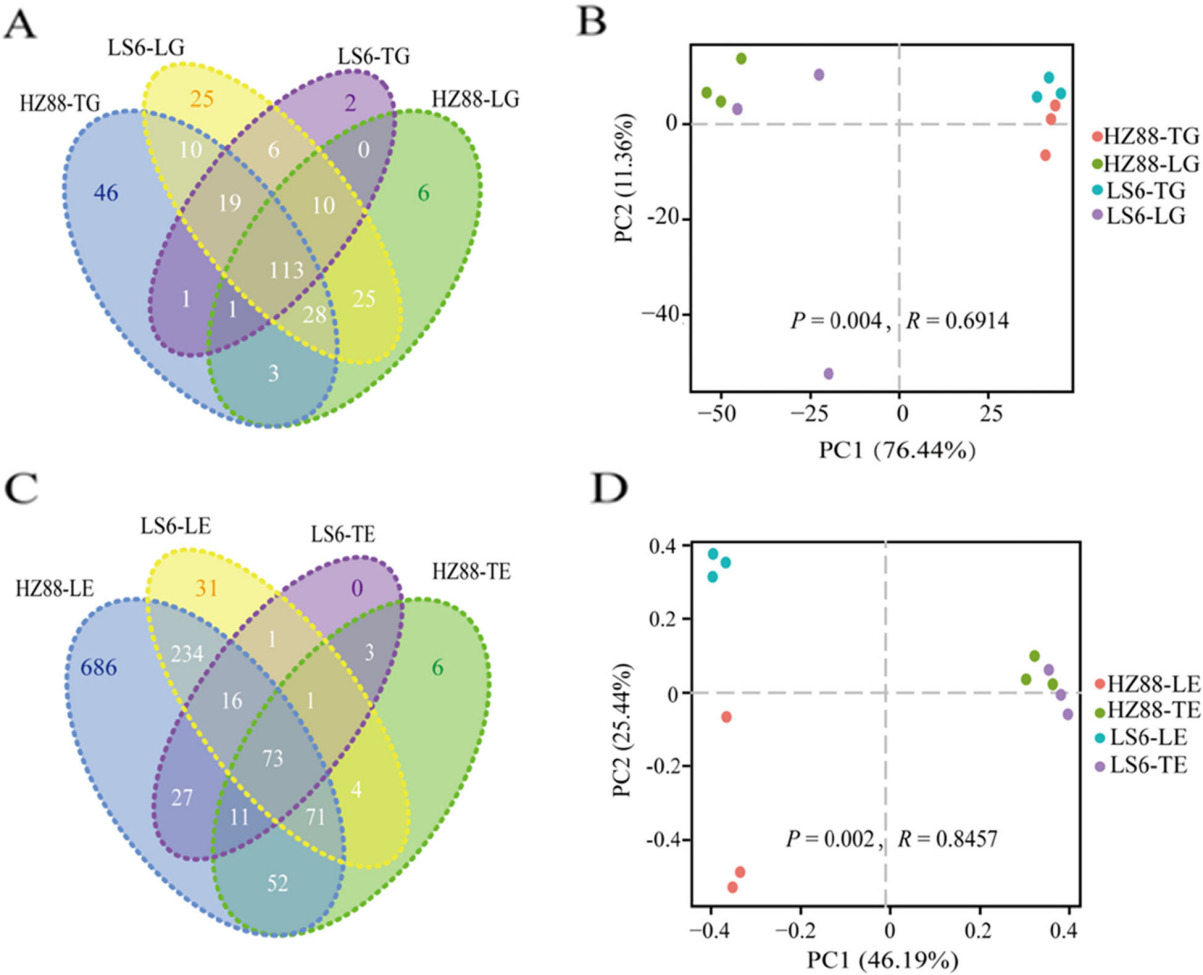
Operational taxonomic units (OTUs) in microbiota of each sample. **a** Venn diagram of OTUs of gut microbiota in the four groups. **b** Venn diagram of OTUs of potato endophytic bacteria in the four groups. **c** Principal coordinate analyses (PCoA)-score plots based on Bray–Curtis dissimilarity analysis for the PTM midgut microbiota. **d** PCoA-score plots based on Bray–Curtis dissimilarity analysis for potato endophytic bacteria. LS6-LG and LS6-TG refer to the midgut samples of PTMs fed on the leaves and tubers of cultivar LS-6, respectively. HZ88-LG and HZ88-TG refer to the midgut samples of PTMs fed on the leaves and tubers of HZ-88, respectively

The results of high-throughput Illumina deep sequencing of the 16S rRNA gene are shown in Additional file 5: Table S1. A total of 4,152,883 reads with a median length of 423 bp (ranging from 416 to 426) were generated from 12 PTM midgut samples. After filtering out low-quality reads, adapters, and overlapping paired-end reads (PEs), 3,101,947 clean tags remained, accounting for 75.00% of the valid tags with an average of 258,496 clean tags (ranging from 199,065 to 326,764). As shown in the Venn diagram (Fig. 2a), a total of 231 operational taxonomic units (OTUs) with 97% identity cutoffs were found. We identified 221 OTUs in HZ88-TG, 186 OTUs in HZ88-LG, 152 OTUs in LS6-TG, 231 OTUs in LS6-LG, and 113 OTUs common to all groups.

A total of 1,936,772 reads with a median length of 445 bp (range from 437 to 445) were generated from 12 potato tuber and leaf samples (Additional file 6: Table S2). After filtering out low-quality reads, adapters, and overlapping PE, 1,777,204 clean tags remained, accounting for 88.99% of the valid tags with an average of 148,100 clean tags (ranging from 69,725 to 244, 755). As a result, a total of 1211 OTUs with 97% identity cutoffs were found, as shown in the Venn diagram in Fig. 2b. We found 1170 OTUs in HZ88-LE, 216 OTUs in HZ88-TE, 132 OTUs in LS6-TE, 494 OTUs in LS6-LE, and 73 OTUs common to all groups.

To assess the sequencing depth and species richness, a rarefaction curve was constructed for each treatment. Most rarefaction curves approached saturation, indicating that our sequencing depth was sufficient to detect the majority of abundant OTUs associated with the midgut contents from leaf- or tuber-fed PTMs of both cultivars (Additional file 1: Figure S1A) and unattacked leaves and tubers from both cultivars (Additional file 1: Figure S1B) .

### α- and β-diversities of midgut symbiotic bacteria of PTM and endophytic bacteria of potato tissues

The α-diversity indices (Table 1) compared bacterial diversities in the midgut of PTM larva feeding solely on either potato leaves or tubers. The ACE and Chao1 indices revealed significant differences in the diversity of the bacterial communities in tuber- and leaf-feeding PTMs living on two potato cultivars. The ACE and Chao1 indices of PTM midgut bacteria after feeding on HZ-88 tubers were significantly higher than those feeding on HZ-88 leaves, while the ACE and Chao1 indices for PTMs feeding on LS-6 tubers were significantly lower than for those feeding on LS-6 leaves. No significant differences were found, based on the Simpson and Shannon indices, in the midgut bacterial communities of tuber- or leaf-feeding PTMs living on two potato cultivars. All diversity indices (except for the ACE index) indicated that the endophytic bacteria in the leaves of both potato cultivars showed significantly higher diversity than those of endophytic bacteria in potato tubers. These results indicated that the diversity of endophytic bacterial species in potato leaves was higher than that in potato tubers.

**Table 1:** α-diversity indices for symbiotic bacteria in PTM midgut and endophytic bacteria in potato tissues

Tissue	Sample names	ACE	Chao1	Simpson	Shannon
PTM midgut	HZ88-TG	196.619	194.751	0.525	1.430
	HZ88-LG	126.164	125.189	0.675	0.801
	*T*-test	*F* = 3.626, *P* < 0.05	*F* = 4.830, *P* < 0.05	*F* = 0.200, *P* > 0.05	*F* = 0.574, *P* > 0.05
	LS6-TG	119.608	119.167	0.414	1.345
	LS6-LG	180.047	173.042	0.424	1.806
	*T*-test	*F* = 3.321, *P* < 0.05	*F* = 8.120, *P* < 0.05	*F* = 1.933, *P* > 0.05	*F* = 2.749, *P* > 0.05
Potato tissues	HZ88-TE	192.854	154.413	0.225	1.714
	HZ88-LE	638.641	643.357	0.057	4.420
	*T*-test	*F* = 0.407, *P* < 0.05	*F* = 0.773, *P* < 0.05	*F* = 0.016, *P* < 0.05	*F* = 6.322, *P* < 0.05
	LS6-TE	278.973	150.715	0.233	1.744
	LS6-LE	312.632	317.977	0.118	3.026
	*T*-test	*F* = 6.463, *P* > 0.05	*F* = 3.961, *P* < 0.05	*F* = 0.239, *P* < 0.05	*F* = 0.267, *P* < 0.05

HZ88-TG refers to the midgut bacteria of PTMs living on the tubers of cultivar HZ-88, and HZ88-LG refers to the midgut bacteria of PTMs living on the leaves of cultivar HZ-88. LS6-TG refers to midgut bacteria of PTMs living on the tubers of potato cultivar LS6, and LS6-LG refers to midgut bacteria of PTMs living on the leaves of potato cultivar LS6. HZ88-TE refers to endophytic bacteria in the tubers of potato cultivar HZ-88, and HZ88-LE refers to endophytic bacteria in the leaves of potato cultivar HZ-88. LS6-TE refers to endophytic bacteria in the tubers of potato cultivar LS-6, and LS6-LE refers to endophytic bacteria in the leaves of potato cultivar LS-6

Principal component analysis (PCoA) compared bacterial compositions in the midgut of PTM larvae after feeding solely on either potato tubers or leaves (Fig. 2c). Significant differences in midgut bacterial compositions were found in the midgut of tuber-feeding and leaf-feeding PTMs (Bray–Curtis analysis of similarities (ANOSIM), *P* = 0.005). Bray–Curtis dissimilarity was used to analyze the two first coordinates, and the cumulative percent of two PCoA estimators explained 87.80% of the sequence diversity. The bacterial composition of the midgut of tuber-feeding larvae formed distinct clusters from those of leaf-feeding larvae. Moreover, results revealed that the midgut bacterial communities in leaf-feeding PTMs living on different potato cultivars were very similar, while they were markedly different in tuber-feeding PTMs living on different potato cultivars.

A significant difference was found for the endophytic bacterial communities in potato tuber and leaf samples, based on the PCoA results (Bray–Curtis ANOSIM, *P* = 0.005; Fig. 2d). The cumulative percentage of two PCoA estimators explained 71.63% of the sequence diversity by the Bray–Curtis dissimilarity. Moreover, no significant difference was found for the endophytic bacteria communities in the tubers or leaves of both potato cultivars. These results demonstrated that the endophytic bacterial communities in the same tissue type were similar between different potato cultivars.

### Composition of microbial communities in the midgut of PTMs feeding on potato tubers and leaves

To further analyze the taxonomy of midgut microbiota, all OTUs were annotated using the Silva database. Eleven phyla were found in the midgut of tuber- and leaf-feeding PTMs living on both cultivars (Additional file 2: FigureS2A). Among them, Actinobacteria, Bacteroidetes, Cyanobacteria, Firmicutes, and Proteobacteria were detected in all midgut samples. Acidobacteria was specifically found in the midgut of tuber-feeding PTMs living on LS-6. Candidate division TM7 was found in the midgut of leaf-feeding PTMs living on both cultivars. Chloroflexi and Nitrospirae were specifically found in the midgut of leaf-feeding PTMs living on LS-6. Deinococcus-Thermus was specifically found in the midgut of tuber- and leaf-feeding PTMs living on HZ-88 potato cultivars. Interestingly, SHA-109 was specifically found in the midgut of tuber- and leaf-feeding PTMs living on LS-6 potato cultivars. In addition, the relative abundances of Proteobacteria, Firmicutes, Cyanobacteria, Bacteroidetes, and Actinobacteria were > 1%. Proteobacteria and Firmicutes were the dominant phylum in all midgut samples. The relative abundance of Proteobacteria was higher than that of other phyla, and ranging from 87.59 to 97.59%, followed by that of Firmicutes bacteria, which ranged from 0.34 to 11.32%.

**Fig. 3:**
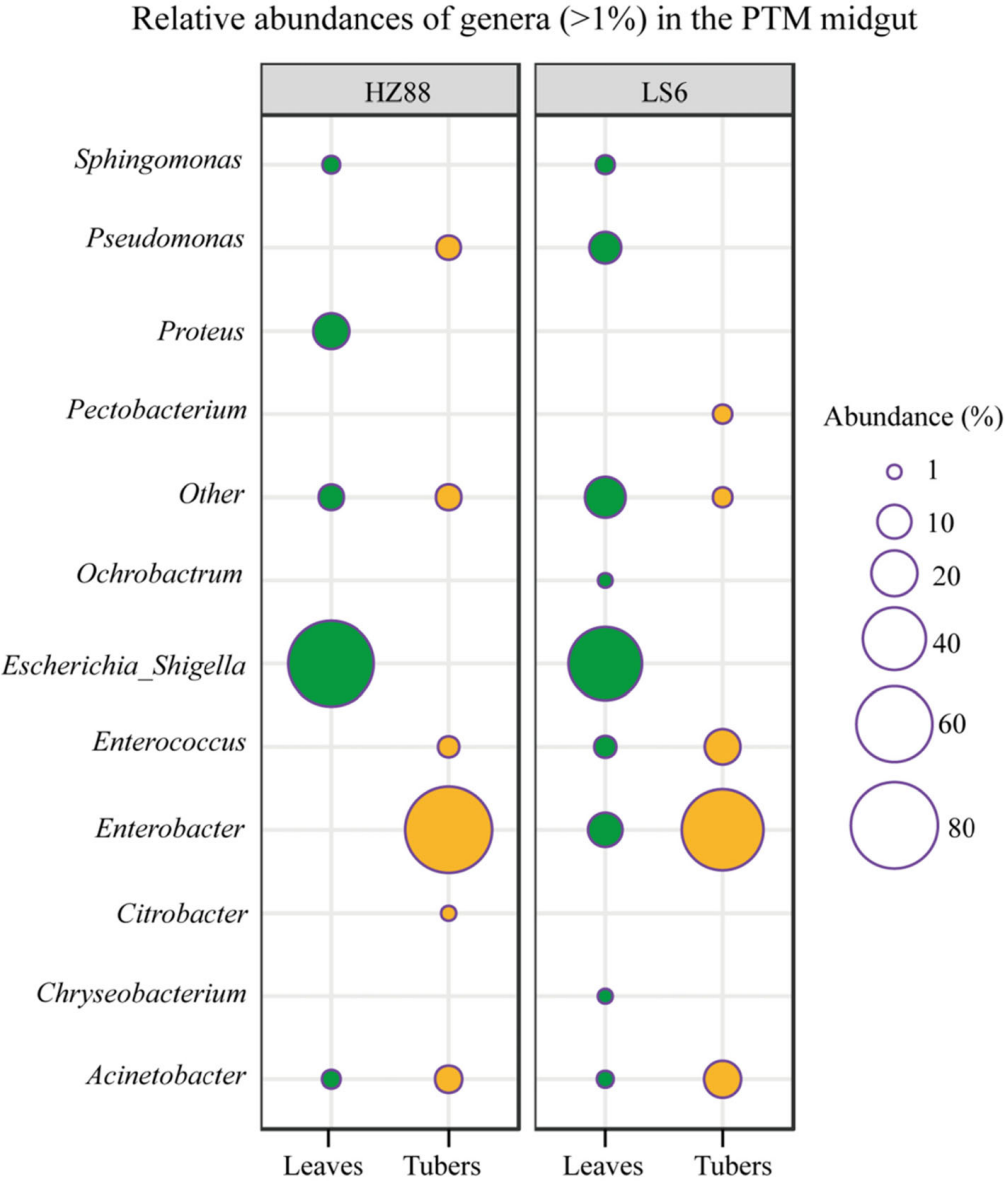
Midgut microbial genera with relative abundance of > 1% in PTMs. The size of the circle indicates the relative abundance of each genus in the midgut PTMs living on leaves and tubers of different potato cultivars. The green circles represent PTMs feeding on leaves, whereas the yellow circles represent PTMs feeding on tubers. HZ88 and LS6 refer to the HZ-88 and LS-6 potato varieties, respectively. The “Others” indicate the all midgut microbial genera with relative abundance of < 1% in PTMs and unannotated genera

Among the intestinal microflora of PTMs, 109 genera were detected in all midgut samples, but only 11 genera displayed a relative abundance of > 1% (Fig. 3 and (Additional file 2: Figure S2B), among which were *Chryseobacterium*, *Enterococcus*, *Ochrobactrum*, *Methylobacterium*, *Sphingomonas*, *Citrobacter*, *Enterobacter*, *Escherichia_Shigella*, *Proteus*, *Acinetobacter*, *Pseudomonas*, and *Pectobacterium*. In addition, 77 and 102 genera were detected in the midgut of leaf-feeding PTMs living on HZ-88 and LS-6, respectively. Seventy-four common genera were detected in the midgut of leaf-feeding PTMs living on either cultivar. The top 4 predominant populations in the midgut of leaf-feeding PTMs living on HZ88 were *Escherichia_Shigella*, *Proteus*, *Acinetobacter*, and *Sphingomonas*, the relative abundances of which were 78.239, 11.503, 2.209, and 1.918%, respectively. The top 8 predominant populations in the midgut of leaf-feeding PTMs living on LS-6 were *Escherichia_Shigella*, *Enterobacter*, *Pseudomonas*, *Enterococcus*, *Sphingomonas*, *Acinetobacter*, *Chryseobacterium*, and *Ochrobactrum*, which showed relative abundances of 55.817, 10.362, 8.573, 3.469, 2.287, 1.643, 1.150, and 1.079%, respectively.

We detected 74 and 70 genera in the midgut of tuber-feeding PTMs living on HZ-88 and LS-6, respectively (Fig. 3). Sixty common genera were detected in midguts of tuber-feeding PTMs living on either cultivar. The top 6 predominant populations in the midgut of tuber-feeding PTMs living on HZ-88 were *Enterobacter*, *Acinetobacter*, *Pseudomonas*, *Enterococcus*, *Sphingomonas*, and *Citrobacter*, which showed relative abundances of 79.795, 5.849, 4.472, 3.154, 1.959, and 1.207%, respectively. The top four predominant populations in the midguts of tuber-feeding PTMs living on LS-6 were *Enterobacter*, *Acinetobacter*, *Enterococcus*, and *Pectobacterium*, which showed relative abundances of 69.929, 12.193, 11.247, and 2.393%, respectively.

In summary, these results indicated that the midgut bacteria was similar after feeding on the same type of tissue. The abundances of *Enterobacter*, *Acinetobacter*, and *Enterococcus* in both tuber-feeding PTM populations were higher than those in both leaf-feeding populations. The abundances of *Escherichia_Shigella* in leaf-feeding populations were higher than that in tuber-feeding populations. Moreover, *Enterobacter* was the dominant genus in the midgut of both tuber-feeding populations, and *Escherichia_Shigella* were the dominant genera in the midgut of both leaf-feeding populations.

### Intestinal microflora differences in the midgut of PTMs feeding on leaves and tubers of different potato cultivars

**Fig. 4:**
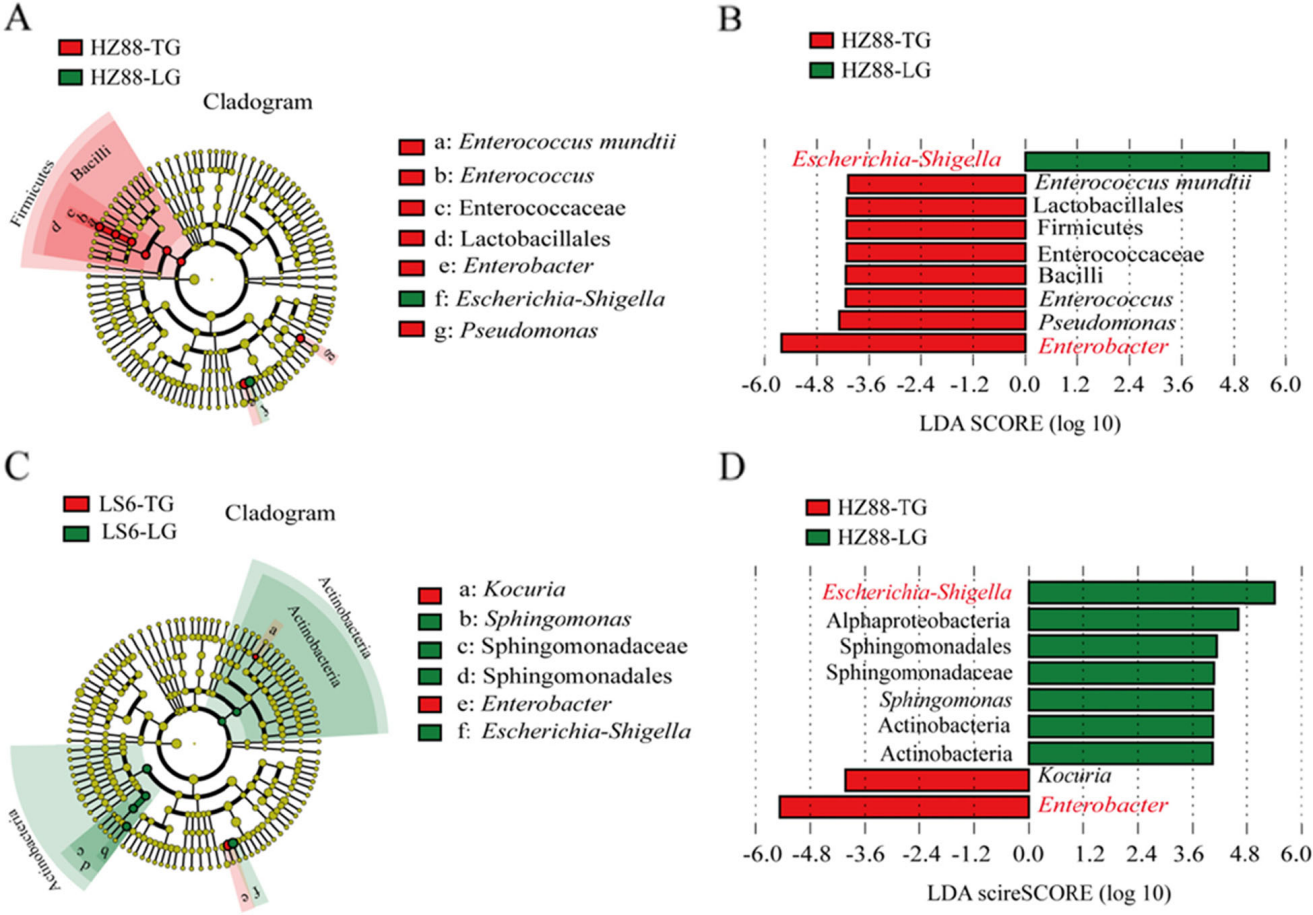
Differences in identified bacterial taxa observed with LEfSe analysis in the midgut of PTMs. **a** Cladogram of bacterial taxa from the midgut of PTMs living on HZ-88 potato leaves (HZ88-LG) and tubers (HZ88-TG). The brightness of each dot is proportional to its effect size. **b** Cladogram of bacterial taxa from the midgut of PTMs living on LS-6 potato leaves (LS6-LG) and tubers (LS6-TG). The brightness of each dot is proportional to its effect size. The brightness of each dot is proportional to its effect size. **c** Histogram of the linear discriminant analysis (LDA) scores computed for differentially abundant bacterial taxa between PTMs fed on HZ-88 tubers (HZ88-TG) and leaves (HZ88-LG). The red bars indicate a negative score, and the green bar represents a positive score. **d** Histogram showing LDA scores computed for differentially abundant bacterial taxa between PTMs fed on LS-6 tubers (LS6-TG) and leaves (LS6-LG). The red bars indicate a negative score, and the green bars indicate a positive score. LS6-LG and LS6-TG refer to the midgut samples of PTMs fed on the leaves and tubers of cultivar LS-6, respectively. HZ88-LG and HZ88-TG refer to the midgut samples of PTMs fed on the leaves and tubers of HZ-88, respectively

To identify differences in bacterial taxa in the midgut microbiota of PTMs, Linear discriminant analysis effect size (LEfSe) analysis was performed on the basis of discriminant analysis (LDA) scores > 4.0 (Fig. 4). Nine bacterial taxa (1 phylum, 1 order, 1 class, 1 family, 4 genera, and 1 species) were distinguished in tuber- and leaf-feeding PTMs living on potato cultivar HZ-88. The Firmicutes phylum; the Lactobacillales order; the Enterococcaceae family; the *Enterococcus*, *Pseudomonas*, and *Enterobacter* genus; and the *Enterococcus mundtii* species were enriched in the midgut of tuber-feeding PTMs living on HZ-88. In contrast, the *Escherichia_Shigella* genera were enriched in the midgut of leaf-feeding PTMs living on HZ-88. Moreover, 9 bacterial taxa (1 phylum, 1 order, 2 class, 1 family, 4 genera, and 1 species) were distinguished in tuber- and leaf-feeding PTMs living on potato cultivar LS-6. The Actinobacteria phylum, the Sphingomonadales order, the Actinobacteria and Alphaproteobacteria classes, the Sphingomonadaceae family, and the *Escherichia_Shigella* and *Sphingomonas* genera were enriched in the midgut of leaf-feeding PTMs living on potato cultivar LS-6, whereas two genera (*Enterobacter* and *Kocuria*) were enriched in the midgut of tuber-feeding PTMs living on potato cultivar LS-6. LEfSe analysis showed that the dominant *Enterobacter* and *Escherichia_Shigella* genera were significantly different from that in the midgut of tuber- and leaf-feeding PTMs living on either potato cultivar.

### Co-relationship between midgut microbiota and the nutrient contents in the leaves and tubers of different potato cultivars

To clarify the relationship between midgut microbiota and the nutrient contents of potato tissues, the nutrient content of the leaves and tubers of both tested potato cultivars were evaluated (Table 2). The nutrient contents in the potato leaves and tubers of the LS-6 and HZ-88 potato cultivars were significantly different. The soluble sugar, crude fiber, protein, and water contents in leaves was significantly higher than those in tubers of both potato cultivars. However, the starch and amino acid contents in the leaves of both potato cultivars were significantly lower than those in the tubers. The crude fiber content in potato leaves was nearly 20-fold higher than that in the tubers, while the starch content in potato tubers was approximately 24-fold higher than that in the leaves.

**Table 2:** Nutrient contents in potato leaves and tubers

Potato variety	Potato organs	Soluble sugars (mg/g)	Starch (%)	Crude fiber (%)	Protein (mg/g)	Amino acid (μmol/g)	Water Content (%)
LS-6	Leaves	10.00 ± 1.07	2.02 ± 0.57	28.37 ± 1.40	34.37 ± 3.02	74.22 ± 15.99	90.91 ± 0.26
	Tubers	3.84 ± 0.72	25.03 ± 5.15	2.38 ± 0.06	8.83 ± 0.68	175.05 ± 42.96	83.23 ± 2.95
	*T*-test	*F* = 0.20, *P* < 0.05	*F* = −12.17, *P* < 0.05	*F* = 14.21, *P* < 0.05	*F* = 6.01, *P* < 0.05	*F* = −1.71, *P* < 0.05	*F* = 10.72, *P* < 0.05
HZ-88	Leaves	7.27 ± 0.42	1.716 ± 0.37	26.23 ± 1.64	42.67 ± 3.24	82.68 ± 11.93	90.57 ± 0.03
	Tubers	1.45 ± 0.16	35.02 ± 4.94	2.63 ± 0.44	7.06 ± 1.89	121.39 ± 11.47	77.19 ± 2.18
	*T*-test	*F* = 2.06, *P* < 0.05	*F* = 8.01, *P* < 0.05	*F* = 2.19, *P* < 0.05	*F* = 1.33, *P* < 0.05	*F* = 0.00001, *P* < 0.05	*F* = 8.88, *P* < 0.05

HZ-88: Hezuo-88 potato cultivar. LS-6: Lishu-6 potato cultivar

Correlations between abundances in intestinal microbial community and the nutrient contents of the leaves and tubers were determined (Table 3). The results showed that the *Enterobacter* and *Citrobacter* abundances correlated positively with the starch and amino acid contents, but correlated negatively with the soluble sugar, crude fiber, protein, and water contents. In contrast, the abundances of *Escherichia_Shigella* and *Ochrobactrum* correlated negatively with the starch and amino acid contents, but correlated positively with the soluble sugar, crude fiber, and protein contents. Of note, the abundance of *Ochrobactrum* was 1% higher than that in HZ-88 tubers, and that of *Citrobacter* was 1% higher than in LS-6 leaves. These results suggested that the microbial community structure correlated significantly with the nutrient content of the tissues and that the PTM midgut abundances of the dominant *Escherichia_Shigella* and *Enterobacter* genera were affected by the nutrient contents of potatoes*.*

**Table 3:** Correlation between abundance of intestinal microflora in PTM midgut and nutrient contents in potato tissue

Nutrient content	*Citrobacter*	*Enterobacter*	*Escherichia_Shigella*	*Ochrobactrum*
Soluble sugars	−0.722**	− 0.878**	0.781**	0.583*
Starch	0.706*	0.928**	−0.842**	−0.480
Crude fiber	−0.618*	−0.953**	0.857**	0.547
Protein	−0.619*	−0.954**	0.865**	0.427
Amino acid	0.306	0.777**	−0.708**	−0.573
Water content	−0.738**	−0.882**	0.797**	0.468

** Significant at the *P* < 0.01 alpha level; *Significant at the *P* < 0.05 alpha level

### Microbial community compositions in the leaves and tubers of different potato cultivars

Endophytic bacteria in potato leaves and tubers was examined to study relationships between midgut microbiota and potato endophytic bacteria. Twenty-eight phyla were found in the leaves and tubers of both potato cultivars (Additional file 3: Figure S3A). All phyla were detected in the leaves of HZ-88, and 9 were detected in its tubers. Sixteen phyla were detected in the leaves of LS-6, and 7 phyla were detected in its tubers. However, only 4 phyla (Proteobacteria, Firmicutes, Bacteroidetes, and Actinobacteria) showed relative abundances of > 1%. Proteobacteria was dominant across all groups and its relative abundance was the highest, ranging from 50.114 to 91.790%.

**Fig. 5:**
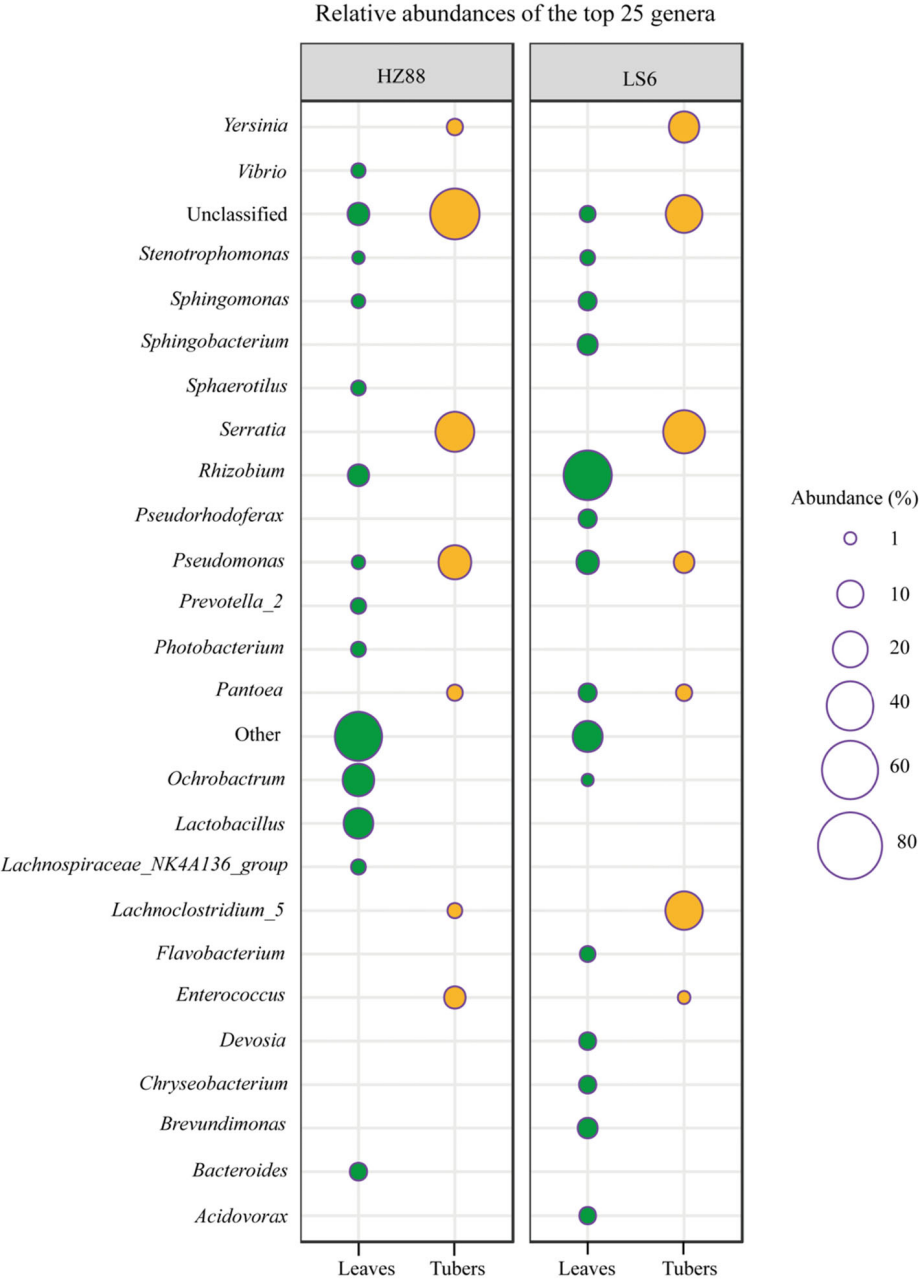
Top 25 genera of endophytic bacteria in leaves and tubers from different potato cultivars. The size of each circle indicates the relative abundance of each genus among the potato endophytic bacteria from different potato cultivars. The green circles indicate endophytic bacteria in potato leaves, whereas the yellow circles indicate endophytic bacteria in potato tubers. HZ88 and LS6 refer to the HZ-88 and LS-6 potato varieties, respectively. The “Others” indicate the total relative abundance of less than top 25 genera and unannotated genera of endophytic bacteria in leaves and tubers from different potato cultivars

A total of 498 genera of endophytic bacteria were detected in all groups (Fig. 5 and Additional file 3: Figure S3B). Among them, 466 and 223 genera were detected in the leaves of HZ-88 and LS-6, respectively. The top 12 predominant populations in the leaves of HZ-88 were *Ochrobactrum*, *Lactobacillus*, *Rhizobium*, *Bacteroides*, *Prevotella_2*, *Photobacterium*, *Lachnospiraceae NK4A136 group*, *Sphaerotilus*, *Vibrio*, *Sphingomonas*, *Pseudomonas*, and *Stenotrophomonas*, and their relative abundances were 15.523, 13.354, 5.707, 3.237, 2.219, 2.002, 1.997, 1.897, 1.729, 1.471, 1.363, and 1.098%, respectively. The top 13 predominant populations in the leaves of LS-6 were *Rhizobium*, *Pseudomonas*, *Sphingobacterium*, *Brevundimonas*, *Pseudorhodoferax*, *Pantoea*, *Sphingomonas*, *Chryseobacterium*, *Devosia*, *Acidovorax*, *Flavobacterium*, *Stenotrophomonas*, and *Ochrobactrum,* and their relative abundances were 41.866, 6.642, 4.913, 4.832, 3.684, 3.631, 3.550, 3.213, 2.996, 2.888, 2.334, 1.969, and 1.001%, respectively.

In addition, 140 and 86 genera were detected in the tubers of HZ-88 and LS-6, respectively (Fig. 5). The top 6 predominant populations in the tubers of HZ-88 were *Serratia*, *Pseudomonas*, *Enterococcus*, *Yersinia*, *Pantoea,* and *Lachnoclostridium 5*, and their relative abundances were 25.442, 17.024, 5.903, 2.624, 2.448, and 1.999%, respectively. The top 6 predominant populations in tubers of LS-6 were *Serratia*, *Lachnoclostridium* 5, *Yersinia*, *Pseudomonas*, *Pantoea*, and *Enterococcus*, and their relative abundance were 30.168, 23.371% ± 16.571, 14.150, 5.214, 2.644, and 1.165%, respectively. It is worth noting that the endophytic bacteria of tubers of both cultivars included many unclassified species, and their relative abundances in tubers were much higher than that in leaves.

These results conclusively demonstrated that the abundances of endophytic bacteria at the genus level in potato leaves and tubers of HZ-88 and LS-6 potato cultivars were similar.

### Difference of endophytic bacteria on leaves and tubers of different potato cultivars

We performed LEfSe analysis based on LDA scores > 4.0 to identify endophytic bacteria on leaves and tubers of both potato cultivars (Additional file 4: Figure S4). We identified 57 distinguished bacterial taxa among the 4 groups. Two endophytic bacteria genera (*Serratia* and *Yersinia*) were significantly abundant in LS-6 tubers. One class (Gammaproteobacteria), one order (Enterobacteriales), and one family (Enterobacteriaceae) were significantly abundant in HZ-88 tubers. Twenty-nine bacterial taxa were significantly abundant in LS-6 leaves, including 4 classes, 6 orders, 8 families, 10 genera, and 1 species. Twenty-three bacterial taxa were significantly abundant in the leaves of HZ-88, including 1 kingdom,1 phylum, 3 orders, 2 classes, 7 families, 6 genera, and 3 species.

### Shared microbiota genera in the midgut of PTMs and potato endophytic bacteria

**Fig. 6:**
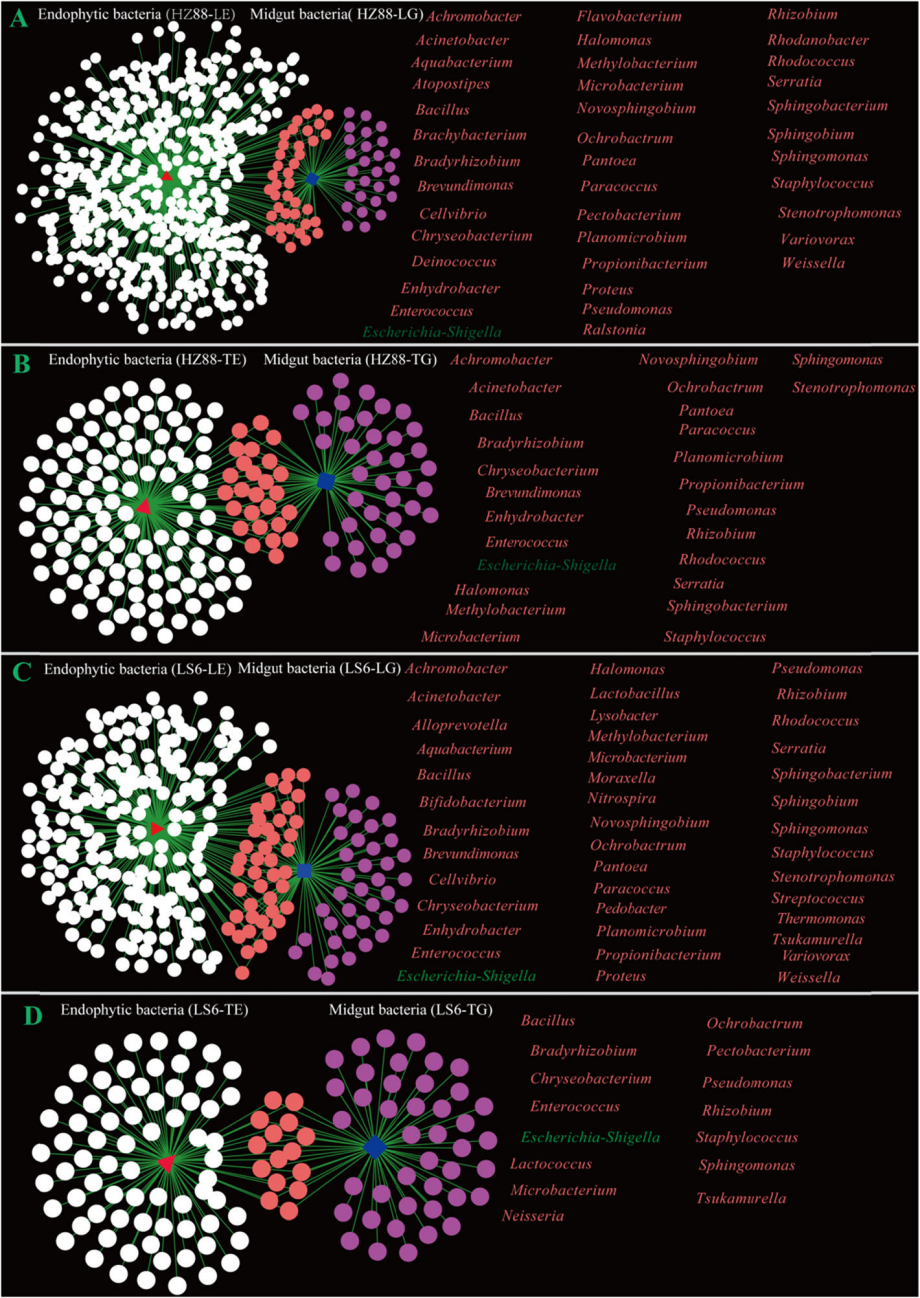
Shared genera between the midgut of PTMs and its host potato endophytic bacteria. **a** Shared genera between midgut microbial genera PTMs and endophytic bacteria genera in the leaves of potato cultivar HZ-88. **b** Shared genera between midgut microbial genera PTMs and endophytic bacteria genera in the tubers of potato cultivar HZ-88. **c** Shared genera between midgut microbial genera PTMs and endophytic bacteria genera in the leaves of potato cultivar LS-6. **d** Shared genera between midgut microbial genera PTMs and endophytic bacteria genera in the tubers of potato cultivar LS-6. HZ88-LE refers to endophytic bacteria in the leaves of potato cultivar HZ-88, and HZ88-TE refers to endophytic bacteria in the tubers of potato cultivar HZ-88. HZ88-LG refers to the midgut bacteria of PTMs living on the leaves of cultivar HZ-88, and HZ88-TG refers to PTMs living on the tubers of cultivar HZ-88. LS6-LE refers to endophytic bacteria in the leaves of potato cultivar LS-6, and LS6-TE refers to endophytic bacteria in the tubers of potato cultivar LS-6. LS6-LG refers to midgut bacteria of PTMs living on the leaves of potato cultivar LS6, and LS6-TG refers to midgut bacteria of PTMs living on the tubers of potato cultivar LS6

The similarities between the midgut microbiota of PTMs and endophytic bacteria were compared (Fig. 6 and Additional file 7: Table S3). Thirty-nine genera were shared by the midgut microbiota of PTMs living on HZ-88 leaves and endophytic bacteria in HZ-88 leaves (Fig. 6a). Twenty-six genera were shared by the midgut microbiota of PTMs living on HZ-88 tubers and endophytic bacteria in HZ-88 tubers (Fig. 6b). Forty-two genera were shared by the midgut microbiota of PTMs living on LS-6 leaves and endophytic bacteria in LS-6 leaves (Fig. 6c). Fifteen genera were shared by the midgut microbiota of PTMs living on LS-6 tubers and endophytic bacteria of LS-6 tubers (Fig. 6d). The *Staphylococcus*, *Bradyrhizobium*, *Enterococcus*, *Escherichia_Shigella*, *Ochrobactrum*, *Microbacterium*, *Pseudomonas*, *Rhizobium*, *Sphingomonas*, and *Bacillus* genera were shared across all samples.

As mentioned above, *Enterobacter* was the dominant microbe in the midgut of both HG-88 and LS-6 tuber-feeding populations, and *Escherichia_Shigella* were the dominant microbes in the midgut of both leaf-feeding populations. Interestingly, *Escherichia_Shigella* were found in the leaves and tubers of both potato cultivars, but *Enterobacter* was not detected in all potato samples. Moreover, the relative abundances of *Escherichia_Shigella* in the leaves and tubers of both potato cultivars were very low, with relative abundances of 1.456% (HZ88-LE), 0.002% (HZ88-TE), 0.019% (LS6-LE), or 0.004% (LS6-TE); while they were very high in the midgut of PTMs living on the leaves of the potato cultivars, with relative abundances of 78.239% (HZ88-LG), compared to only 0.141% in the HZ-88 tubers, and 55.817% (LS6-LG), compared to only 0.023% in the LS-6 tubers. Thus, the relative abundances of *Escherichia_Shigella* in the midgut of PTMs increased only after feeding on potato leaves. In addition, the relative abundance of *Rhizobium* in potato leaves was high, although it was low in the midgut of leaf-feeding PTMs of both potato cultivars.

## Discussion

Most of the life parameters of PTMs living on potato leaves or tubers were similar, indicating that PTMs are well-adapted to both types of tissues. Previous findings indicated that potatoes contain a substantial amount of the glycoalkaloids α-chaconine and α-solanine, which show insecticidal properties [28, 29]. The difference of pupa weights and survival rates of PTM larvae feeding on both tissue types in this study might have been caused by differences in nutrient contents or defensive secondary compounds present in leaves and tubers.

The intestinal microbiota plays key roles in digestion, detoxification, immune protection, and insect development [7, 30]. Recent findings suggested that the diversity and population structure of midgut bacteria in lepidopteran herbivores can be influenced by a wide range of environmental factors [21]. Among them, the host plant species plays a driving role in shaping midgut bacterial populations [29, 31]. However, it remains unclear how the midgut bacteria of lepidopteran herbivores are affected by the consumption of specific plant tissues. Because the PTM damages both the leaves and tubers of potato plants, we analyzed the symbiotic bacteria of PTM midguts and the endophytic bacteria of potato tissues. The nutrients of potato leaves and tubers were also measured. The enrichment of specific bacterial communities in the midgut of PTMs feeding on different potato tissues might be related to the different nutrient levels in tissues consumed by the insect. However, PTM populations from different locations is or isn’t the same rules with further experience.

In this study, the α-diversity indices of tuber- and leaf-feeding PTMs living on two potato cultivars indicated that differences occurred in the midgut bacterial species after feeding on either potato cultivar. However, the PCoA results showed that midgut bacterial communities in leaf-feeding PTMs or tuber-feeding PTMs were very similar after feeding on different potato cultivars, indicating the dominant bacterial species in leaf-feeding or tuber-feeding PTMs were very similar. At the phylum level, Proteobacteria and Firmicutes were the predominant phyla in the midgut of PTM larvae living on the potato leaves and tubers of both cultivars. These findings were similar to those reported in other studies [32, 33]. Therefore, the predominance of these two phyla in the midgut of insect larvae was likely the result of long-term co-evolution in insects. At the genus level, *Enterobacter* was the dominant genus in the midguts of tuber-feeding PTMs, and *Escherichia_Shigella* were dominant in the midgut of leaf-feeding PTMs. The results at the genus level indicated that the bacterial community composition in the PTM midgut was influenced by the tissues consumed, owing to their different nutrient contents. Some *Enterobacteriaceae* genera showed different prevalences in PTMs living on different tissues. The *Enterobacteriaceae* present in the midgut of insects are capable of degrading carbohydrates, cellulose, and starch, which comprise the predominant components of plants [34, 35]. Thus, it was not surprising that the abundances of *Escherichia_Shigella* correlated positively with the crude fiber content in potato leaves. However, the degradative functions of midgut microbial *Enterobacter* and *Escherichia_Shigella* sp. have not been proven. Thus, future research is needed to determine whether these dominant midgut microbial species can degrade the key components of potato leaves and tubers. In addition, some studies have shown that gut microbiota can mediate resistance to chemical insecticide [14–16], while others have shown that entomopathogenic fungi interacts with the gut microbiota to accelerate pest mortality [36–38]. So, different gut microbiota leaf-feeding between tuber-feeding PTMs should be taken a full consideration for integrated management of this pest.

*Escherichia_Shigella* represented the dominant bacterial genera in the midgut of leaf-feeding PTMs, and the genera were also detected at a low levels among endophytes in potato leaves and tubers. The abundances of *Escherichia_Shigella* in potato tubers and in the midgut of tuber-feeding PTMs were similar. Moreover, *Escherichia_Shigella* abundances showed significant positive correlation with the dominant crude fiber content of potato leaves and negatively correlated with the dominant soluble starch content of potato tubers. Therefore, the potato endophytic *Escherichia_Shigella* might be established in the midgut of leaf-feeding PTMs during leaf-feeding and may play an important role for the ability of PTMs to digest leaves. However, no corroborating reports on the function of *Escherichia_Shigella* in insects have been published to date.

*Enterobacter* and *Citrobacter* are known to play important roles in insects. Broderick et al. found that *Bacillus thuringiensis* can kill larvae of the gypsy moth when endogenous midgut bacteria *Enterobacter* are present [39]. Sharon et al. found that the mating preference of *Drosophila melanogaster* was affected by some species of *Enterobacter* [17]. *Enterobacter* in the midgut of Colorado potato beetles (*Leptinotarsa decemlineata*) was primarily responsible for suppressing plant defenses [40]. Some *Citrobacter* sp. in the pest fruit fly *Bactrocera dorsalis* can mediate insecticide resistance [14]. In the midgut of *Bactrocera cucurbitae*, *Citrobacter* sp. are responsible for the attraction of adults [41]. *Citrobacter* sp. in the larval gut of the white grub beetle (*Lepidiota mansueta*) and some *Citrobacter* sp. and *Enterobacter* sp. in the gut of the red palm weevil (*Rhynchophorus ferrugineus*) can degrade cellulose [42, 43]. However, no reports have been published to date showing that *Citrobacter* sp. and *Enterobacter* sp. in insects can degrade starch. In this study, *Enterobacter* and *Citrobacter* correlated positively with the starch and amino acid contents, and were the dominant genera in tuber-feeding PTMs. Starch is the most abundant nutrient content of tubers; thus, *Enterobacter* and *Citrobacter* may help PTMs degrade starch. Moreover, *Enterobacter* and *Citrobacter* were not detected in the leaves and tubers of either potato cultivar, indicating that they were not potato endophytes. Thus, these two genera were specific bacteria in the PTM population and may play a key role in adaptation to the nutrient contents of the diet. Little is known, however, regarding these two genera as intestinal microorganisms in PTM or how they spread in PTM populations.

Diet is a very important factor in shaping the gut microbiota of insects [31, 44]. Plant endophytic bacteria might exist in plant-feeding insects and help regulate insect development [20, 45]. In our study, PTM midgut microbes and potato endophytic communities showed significant differences. For instance, the number of potato endophytic bacterial species was higher than that of PTM midgut bacterial species. However, we also found many common genera between PTM midgut microbes and potato endophytic bacteria. The dominant genera *Chryseobacterium*, *Enterococcus*, *Escherichia_Shigella*, *Ochrobactrum*, *Rhizobium*, *Sphingomonas*, and *Pseudomonas* were shared across all samples tested. A limitation of this study is that we generated no conclusive evidence that potato endophyte bacteria can become established in the midgut of PTM larvae. However, the PTM larvae were starved for 24 h before the midgut microbiota was analyzed to avoid residual endophyte bacteria in potato tissues. In addition, some dominant and non-dominant endophyte bacteria from potato tissues could not be detected in the midgut of PTMs. These considerations suggest that endophytes of potato tissues may be established in the midgut of PTM larvae.

## Conclusions

In this study, we evaluated the development of PTMs on leaves and tubers of two potato cultivars, HZ-88 and LS-6, and characterized differences in their midgut intestinal microbes. Our results showed a significant difference between midgut bacterial communities of PTMs living on the leaves and tubers of potatoes. The adaptivity of PTMs to different nutrient contents of potato leaves and tubers was potentially caused by the co-evolution of *Escherichia_Shigella* and *Enterobacter*. In addition, some dominant bacterial genera in the PTM midgut were shared with the endophytic genera of host potato leaves and tubers. We provide evidence that a portion of the intestinal microbes of PTMs may be derived from potato endophytic bacteria. This study provides new insights into the adaptation of PTMs to different nutrient contents of potato leaves and tubers and offers a better understanding of the relationship between potato endophytic bacteria and the gut microbiota of PTMs, which may offer support for integrated management of this worldwide pest.

## Methods

### Rearing potato plants and PTMs

In this study, we used two potato cultivars, Hezuo-88 (HZ-88) and Lishu-6 (LS-6), which are provided state key laboratory for conservation and utilization of bio-resources in Yunnan, college of plant protection, Yunnan Agricultural University and commonly cultivated in the Yunnan province of southwestern China. Potatoes were planted in a greenhouse and grown at a temperature of 25 ± 3 °C 16 h light: 8 h dark, and 60 ± 5 relative humidity. A large number of potatoes of both cultivars were grown continuously for our experiments. Fresh tubers from both potato cultivars were collected from potato plants grown under the same conditions.

Following modifications of Yuan et al.(2019) and Rondon et al. (2009) protocols [46, 47], the initial population of PTMs was collected in 2014 from infested potato plants in potato fields in Xuanwei City, Yunnan province in southwestern China (N26°05′52.3′′, E104°04′27.5′′). The stock culture of the initial population was reared on potato tubers (LS-6) and maintained in a breeding cage at room temperature of 25 ± 3 °C,16 h light: 8 h dark, and 60 ± 5 relative humidity. The plexiglass-case breeding cages (W × L × H = 30 cm × 30 cm × 40 cm) were covered with a fine nylon mesh, leaving the left and right sides open for ventilation. The bottom of the breeding cage was covered with a 5 cm layer of sand for pupation. To collect PTM eggs of the same age, 20 newly emerged males and 30 female moths were paired and kept in plastic octagonal bottles (WxH = 8 cm × 10 cm) covered with a fine mesh gauze. Sterile water was added to a piece of filter paper placed on the mesh gauze to provide an oviposition site for the mated adult moths. The moths laid eggs on the filter paper, the filter paper was removed after 12 h, and the eggs were used in our experiments. The eggs were maintained in the presence of potato leaves and tubers from cultivar LS-6 or HZ-88, until hatching. Potato plants were placed in nylon mesh cages (W × L × H = 1 m × 1 m × 1.2 m), and tubers were placed in octagonal bottles (W × H = 8 cm × 10 cm). All tests were initiated after two generations of separately rearing PTMs on leaves and tubers of the two potato cultivars.

### PTM development

The total development period and mortality of PTMs feeding on leaves and tubers of two potato cultivars were evaluated. To estimate the development time and survival of PTM eggs, pieces of filter paper containing approximately 100 eggs laid on each potato cultivar were placed in separate petri dishes (9 cm diameter), and the number of hatched eggs was observed and recorded daily. Newly hatched larvae of the same age were removed from potato leaves and tubers. Approximately 100 larvae fed on the leaves and tubers of each cultivar. Development of larvae was recorded daily until pupation occurred. Larvae normally abandon tubers and leaves before pupation. Fifty newly pupated pupae were collected after each treatment and weighed, and their pupal periods were also recorded. To analyze total fecundity, 30 male–female pairs of newly emerged adult moths were used. Each pair was placed in a clear cylindrical glass box (W × H = 3 cm × 5 cm) and covered with two-layer fine mesh gauze to provide an oviposition site. The number of eggs laid on the mesh gauze was recorded daily and the mesh gauze was replaced after each egg count. All adult moths were then placed in a new clear cylindrical glass box while the number of eggs was recorded. Daily monitoring continued until the adult moths died. The sex ratio of the adults feeding on leaves or tubers from either potato cultivar was recorded.

### Midgut sample and potato organ collection, and DNA extraction

The midgut contents were collected from 50 4th-instar larvae from feeding leaves of LS-6 (LS6-LG) or HZ-88 (HZ88-LG) and tubers of LS-6 (LS6-TG) or HZ-88 (HZ88-TG), respectively, that were withheld food for 24 h. The larvae were surface-sterilized with 75% ethanol for 90 s and rinsed with sterile deionized water. After surface sterilization, the larvae were dissected under a dissecting microscope using sterile tools to remove midgut tissues, and the midgut contents were homogenized in 1 mL of sterile deionized water [35]. Three replicates were analyzed for each treatment.

Total bacterial DNA from the larval PTM midgut was extracted using the QIAamp DNA Stool Mini Kit (Qiagen, Hilden, Germany) according to the manufacturer’s protocol, with the following changes. To effectively lyse the bacterial cells, the midgut contents were placed in liquid N_2_ and thawed at 37 °C prior to cell lysis. After adding C1 solution (a component of the kit), each sample was completely homogenized by vortexing for 20 s. The subsequent steps were performed according to the manufacturer’s protocol. The DNA products were verified by electrophoresis on 0.8% agarose gels. DNA yields were measured using a NanoDrop ND-1000 spectrophotometer (Thermo Scientific, Wilmington, USA).

Leaves from non-attacked LS-6 (LS6-LE) and HZ-88 (HZ88-LE) cultivars and tubers from non-attacked LS-6 (LS6-TE) and HZ-88 (HZ88-TE) cultivars, planted in similar environments, were collected. The surface soil was washed off the leaves and tubers with sterile water. Prior to DNA extraction, the leaves and tubers were surface-sterilized by successive washing with ddH_2_O for 30 s, 95% ethanol for 1 min, 6% NaOCl for 15 min, and 100% ethanol for 30 s, and then rinsed 3x with sterile ddH_2_O, as described previously [23]. Subsequently, total plant endophyte bacterial DNA was extracted using the PowerSoil® DNA Isolation Kit (MO BIO Laboratories, Carlsbad, USA) according to the manufacturer’s protocol. The extracted DNA was analyzed as described in the above procedures for bacterial DNA.

### PCR amplification and sequencing

To amplify the V3–V4 region of the 16S rRNA genes of PTM midgut bacteria for Illumina deep sequencing, PCR was performed using the universal primers [48]. 338F (5′-ACTCCTACGGGAGGCAGCA-3′) and 806R (5′-GGACTACHVGGGTWTCTAAT-3′) in a total reaction volume of 20 μL, consisting of 13.25 μL H_2_O, 2.0 μL 10× PCR Ex Taq Buffer, 0.5 μL DNA template (100 ng/mL), 1.0 μL 338F (10 mmol/L), 1.0 μL 806R (10 mmol/L), 2.0 μL dNTPs, and 0.25 μL Ex Taq (5 U/mL). After an initial denaturation at 95 °C for 5 min, amplification was performed with 30 cycles of incubation for 30 s at 95 °C, 20 s at 58 °C, and 6 s at 72 °C, followed by a final extension at 72 °C for 7 min. The amplified products were separated by 1.0% agarose gel electrophoresis and recycled from the gel. Library construction and sequencing were performed by Beijing Biomarker Technologies Co., Ltd. (Beijing, China).

The above-mentioned universal PCR primers used with midgut bacteria DNA amplified the 16S rRNA sequence of potato chloroplasts, so the special primers 335F (5′-CADACTCCTACGGGAGGC-3′) and 769R (5′-ATCCTGTTTGMTMCCCVCRC-3′) were used to avoid such amplification. PCR was performed using a protocol similar to the method described above.

### Bioinformatics analysis

The midgut bacteria communities and endophytic bacteria were subjected to the same analytical procedures. First, paired-end reads were merged using FLASH (v1.2.7, http://ccb.jhu.edu/software/ FLASH/) to obtain the raw tags [49]. The raw tags were then filtered and clustered in the next steps. The sequences of the merged tags were compared to the primer sequences, and tags with more than six mismatches were discarded using the FASTX-Toolkit [50]. Tags with an average quality score < 20 in a 50-base pair (bp) sliding window were truncated using Trimmomatic (http://www.usadellab.org/cms/? page=trimmomatic) [51], and tags shorter than 300 bp were removed. We identified possible chimeras by employing UCHIME [52], a tool included in mothur (http://drive5.com/uchime) [53]. The de-noised sequences were clustered using the QIIME UCLUST module, and tags with ≥97% similarity were regarded as operational taxonomic units (OTUs) [54, 55]. Taxonomy was assigned to all OTUs by searching against the Silva databases (Release 119, http://www.arb-silva.de), using the RDP classifier within QIIME [56, 57].

### Determination of nutrients in leaves and tubers

The soluble protein and free amino acid contents of potato leaves and tubers were measured using kits A045–2 (based on Coomassie blue staining) and A026 (based on ninhydrin colorimetry) (Nanjing Jiancheng Biological Engineering Co., Ltd.), respectively. The soluble sugar and starch contents of potato leaves and tubers were measured as described previously [58]. When performing the tests mentioned above, the optical density values of the reaction mixtures were measured using an ultraviolet–visible spectrophotometer (UV-1800PC; Shanghai Mapade Instruments Co., Ltd., Shanghai, China). The crude fibers were determined as previously described [59].

### Statistical analyses

The differences between two groups were compared using Student’s *T*-test in R (version 3.20; The R Project for Statistical Computing, http://www.R-project.org), with *P* < 0.05 considered a significant difference. Correlations between the intestinal microbial community structure and nutrient contents were analyzed using the R Project cor () function. *R*-value of Pearson Correlation Coefficient represented the relevance of two groups, *r* < 0 was considered as a negative correlation, and *r* > 0 was considered a positive correlation. LEfSe analysis [60] was used to reveal abundance differences of bacterial taxa from different midgut samples and potato tissues at all taxonomic levels. The linear discriminant analysis (LDA) score > 4.0 was thought to be significant by default.

## Supplementary information

**Additional file 1: Figure S1.** Rarefaction curves used to estimated richness (at 97% similarity) in samples.

**Additional file 2: Figure S2.** Comparison of > 1% genus of midgut samples in PTMs living on leaves and tubers.

**Additional file 3: Figure S3.** Comparison of endophytic bacteria in leaves and tubers in two potato varieties.

**Additional file 4: Figure S4.** LEfSe identified significantly differentiated endophytic bacterial taxa between leaves and tubers of two potato varieties.

**Additional file 5: Table S1.** Number of analyzed 16S rRNA gene sequences in the midgut microbiota of PTMs.

**Additional file 6: Table S2.** Number of analyzed 16S rRNA gene sequences of endophytic bacteria in leaves and tubers.

**Additional file 7: Table S3.** The relative abundance of shared genera in different samples.

## Data Availability

The data reported in this manuscript were deposited in the NCBI Sequence Read Archive (SRA) as accession numbers SRR5942164 to SRR5942238 under PRJNA 398438.

## Abbreviations

LDA: Linear discriminant analysis
LEfSe: Linear discriminant effect size analysis
OTUs: Operational taxonomic units
PcoA: Principal component analysis
PEs: Paired-end reads
PTM: Potato Tuber Moth
