# Supplementary material for: Midgut microbiota diversity of potato tuber moth associated with potato tissue consumed
Source: BMC Microbiol. 2020 Mar 11;20:58. doi: 10.1186/s12866-020-01740-8 (PMC7066784; doi:10.1186/s12866-020-01740-8)

**Additional file 1:** **Fig.S1.** Rarefaction curves used to estimated richness (at 97% similarity) in samples.

(**A**) Leaf- and tuber-fed PTM midgut samples. HZ88-TG refers to PTMs living on the tubers of cultivar HZ-88, HZ88-LG refers to the midgut bacteria of PTMs living on the leaves of cultivar HZ-88. LS6-TG refers to midgut bacteria of PTMs living on the tubers of potato cultivar LS6, and LS6-LG refers to midgut bacteria of PTMs living on the leaves of potato cultivar LS6. (**B**) Unattacked potato leaf and tuber samples. HZ88-LE refers to endophytic bacteria in the leaves of potato cultivar HZ-88, and HZ88-TE refers to endophytic bacteria in the tubers of potato cultivar HZ-88. LS6-LE refers to endophytic bacteria in the leaves of potato cultivar LS-6, and LS6-TE refers to endophytic bacteria in the tubers of potato cultivar LS-6.


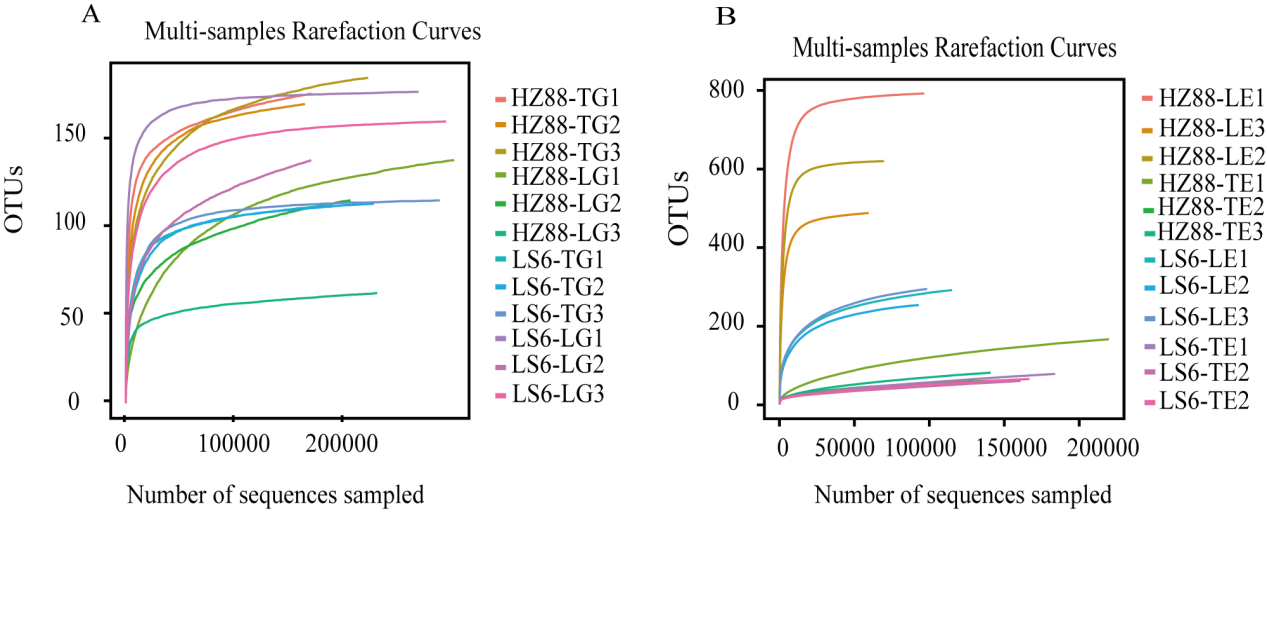

Supplement: Supplementary file 1 — Additional file 1: Figure S1. Rarefaction curves used to estimated richness (at 97% similarity) in samples. [file 12866_2020_1740_MOESM1_ESM.docx]
