# Supplementary material for: Midgut microbiota diversity of potato tuber moth associated with potato tissue consumed
Source: BMC Microbiol. 2020 Mar 11;20:58. doi: 10.1186/s12866-020-01740-8 (PMC7066784; doi:10.1186/s12866-020-01740-8)

**Additional file 2: Fig.S2.** Comparison of > 1% genus of midgut samples in PTMs living on leaves and tubers.

(**A**) Phylum relative abundance. The “Others” indicate the all midgut microbial phylum with relative abundance of <1% in PTMs midgut samples. (**B**) Genus relative abundance. HZ88-TG refers to the midgut bacteria of PTMs living on the tubers of cultivar HZ-88, HZ88-LG refers to the midgut bacteria of PTMs living on the leaves of cultivar HZ-88. LS6-TG refers to midgut bacteria of PTMs living on the tubers of potato cultivar LS6, and LS6-LG refers to midgut bacteria of PTMs living on the leaves of potato cultivar LS6. The “Others” indicate the all midgut microbial genera with relative abundance of <1% and unannotated genera in PTMs midgut samples.


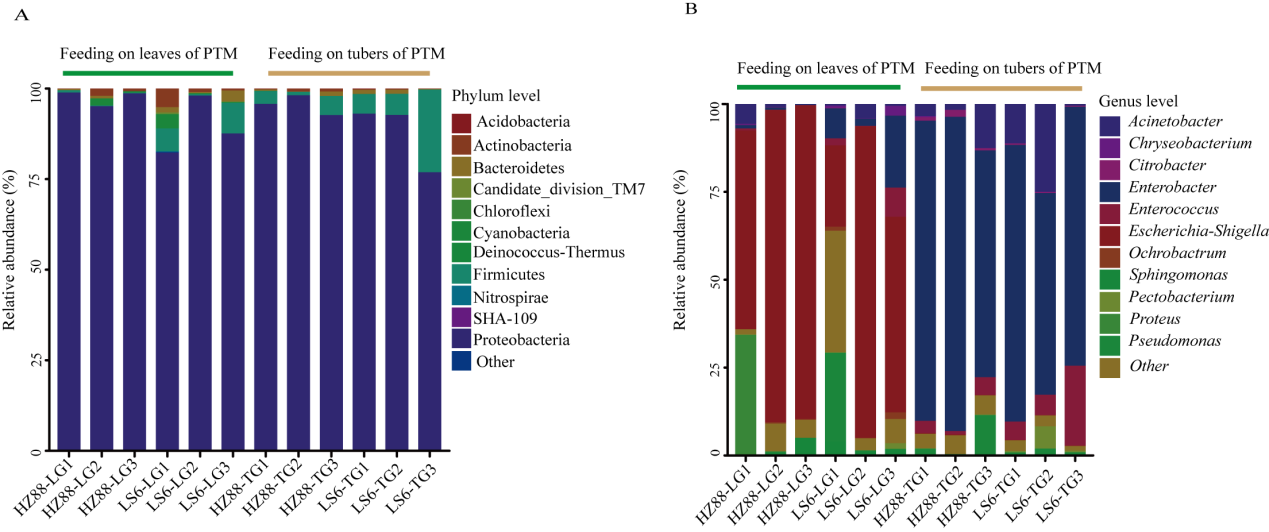

Supplement: Supplementary file 2 — Additional file 2: Figure S2. Comparison of > 1% genus of midgut samples in PTMs living on leaves and tubers. [file 12866_2020_1740_MOESM2_ESM.docx]
