# Supplementary material for: Midgut microbiota diversity of potato tuber moth associated with potato tissue consumed
Source: BMC Microbiol. 2020 Mar 11;20:58. doi: 10.1186/s12866-020-01740-8 (PMC7066784; doi:10.1186/s12866-020-01740-8)

**Additional file 3: Fig.S3.** Comparison of endophytic bacteria in leaves and tubers in two potato varieties.

(**A**) Phylum relative abundance. The “Others” indicate the total relative abundance of less than top 15 phylum of endophytic bacteria in leaves and tubers from different potato cultivars. (**B**) Genus relative abundance. HZ88-LE refers to endophytic bacteria in the leaves of potato cultivar HZ-88, and HZ88-TE refers to endophytic bacteria in the tubers of potato cultivar HZ-88. LS6-LE refers to endophytic bacteria in the leaves of potato cultivar LS-6, and LS6-TE refers to endophytic bacteria in the tubers of potato cultivar LS-6. The “Others” indicate the total relative abundance of less than top 25 genera and unannotated genera of endophytic bacteria in leaves and tubers from different potato cultivars.


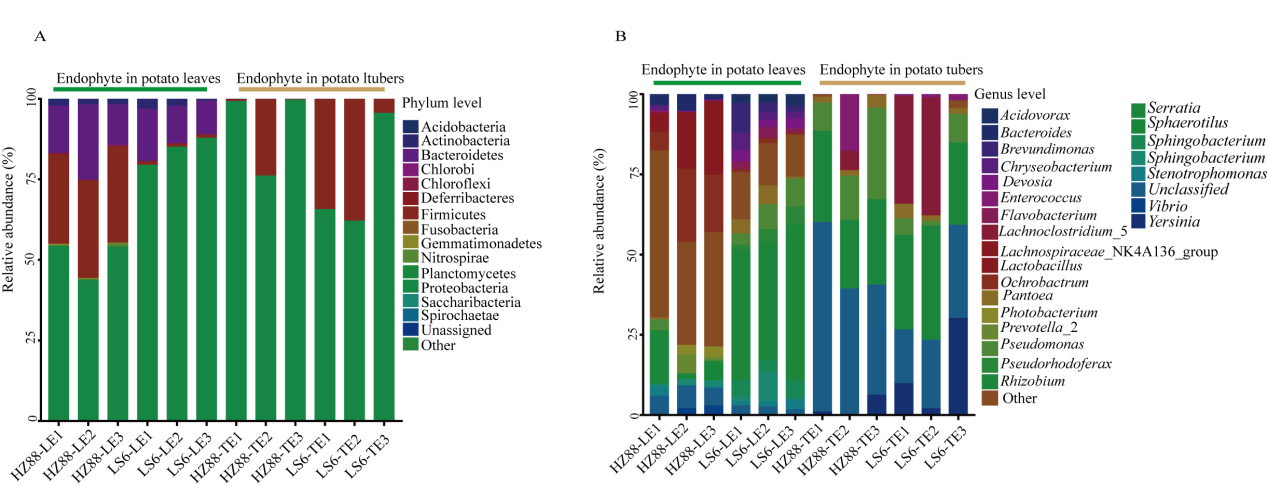

Supplement: Supplementary file 3 — Additional file 3: Figure S3. Comparison of endophytic bacteria in leaves and tubers in two potato varieties. [file 12866_2020_1740_MOESM3_ESM.docx]
