# Supplementary material for: Midgut microbiota diversity of potato tuber moth associated with potato tissue consumed
Source: BMC Microbiol. 2020 Mar 11;20:58. doi: 10.1186/s12866-020-01740-8 (PMC7066784; doi:10.1186/s12866-020-01740-8)

**Additional file 4: Fig.S4.** LEfSe identified significantly differentiated endophytic bacterial taxa between leaves and tubers of two potato varieties.

(**A**) Cladogram of differentially abundant bacterial taxa between tubers and leaves of both potato varieties. The brightness of each dot is proportional to its effect size. (B) Histogram of the linear discriminant analysis (LDA) scores computed for differentially abundant bacterial taxa between tubers and leaves of both potato varieties. The enriched taxa from HZ-88 tuber endophytic bacteria (HZ88-TE) are indicated with a blue LDA score, and enriched taxa from leaf endophytic bacteria (HZ88-LE) are indicated with a yellow LDA score. The enriched taxa from LS-6 tuber endophytic bacteria (LS6-TE) are indicated with a red LDA score, and enriched taxa from leaf endophytic bacteria (LS6-LE) are indicated with a green LDA score. HZ88-LE refers to endophytic bacteria in the leaves of potato cultivar HZ-88, and HZ88-TE refers to endophytic bacteria in the tubers of potato cultivar HZ-88. LS6-LE refers to endophytic bacteria in the leaves of potato cultivar LS-6, and LS6-TE refers to endophytic bacteria in the tubers of potato cultivar LS-6.


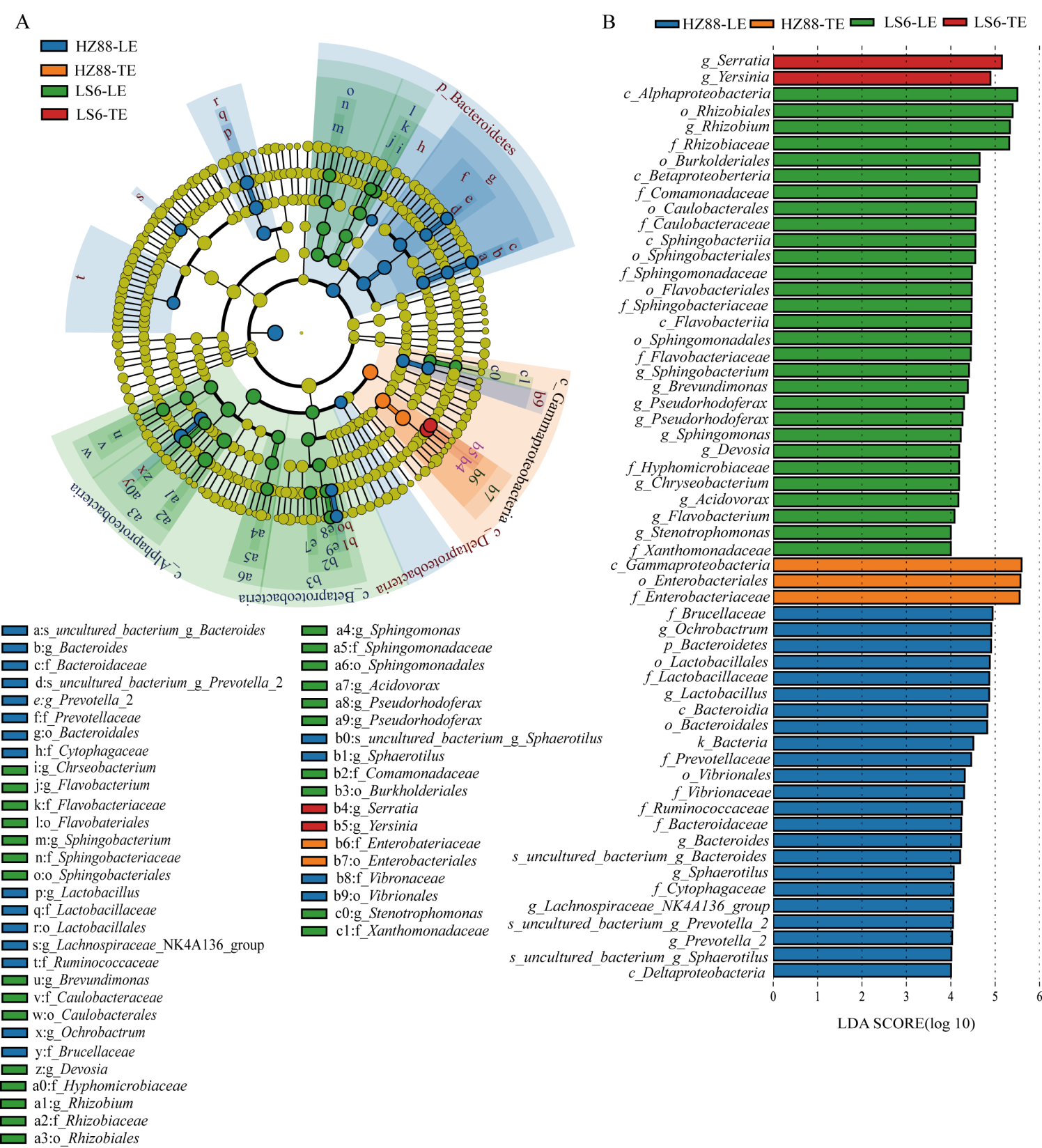

Supplement: Supplementary file 4 — Additional file 4: Figure S4. LEfSe identified significantly differentiated endophytic bacterial taxa between leaves and tubers of two potato varieties. [file 12866_2020_1740_MOESM4_ESM.docx]
