# Supplementary material for: Midgut microbiota diversity of potato tuber moth associated with potato tissue consumed
Source: BMC Microbiol. 2020 Mar 11;20:58. doi: 10.1186/s12866-020-01740-8 (PMC7066784; doi:10.1186/s12866-020-01740-8)
Supplement: Supplementary file 5 — Additional file 5: Table S1. Number of analyzed 16S rRNA gene sequences in the midgut microbiota of PTMs. [file 12866_2020_1740_MOESM5_ESM.docx]

**Additional file 5: Table S1.** Number of analyzed 16S rRNA gene sequences in the midgut microbiota of PTMs

| Sample_ID | PE_Reads | Clean_Tags | AvgLen (bp) | GC (%) | Q20 (%) | Q30 (%) | Effective (%) |
| --- | --- | --- | --- | --- | --- | --- | --- |
| HZ88-TG1 | 310843 | 236651 | 426 | 55.73 | 91.71 | 78.74 | 76.13 |
| HZ88-TG2 | 260421 | 199065 | 426 | 55.96 | 91.68 | 78.67 | 76.44 |
| HZ88-TG3 | 381118 | 287931 | 426 | 54.60 | 91.45 | 78.27 | 75.55 |
| HZ88-LG1 | 400898 | 313762 | 425 | 54.48 | 91.88 | 79.08 | 78.26 |
| HZ88-LG2 | 281145 | 217077 | 423 | 55.38 | 91.87 | 78.92 | 77.21 |
| HZ88-LG3 | 315997 | 239605 | 423 | 55.35 | 91.99 | 79.19 | 75.83 |
| LS6-TG1 | 282308 | 212525 | 424 | 55.23 | 90.87 | 77.02 | 75.28 |
| LS6-TG2 | 310267 | 245059 | 424 | 54.34 | 91.63 | 78.58 | 78.98 |
| LS6-TG3 | 393554 | 312624 | 424 | 55.22 | 91.64 | 78.60 | 79.44 |
| LS6-LG1 | 386708 | 305182 | 416 | 54.35 | 92.09 | 79.37 | 78.92 |
| LS6-LG2 | 409367 | 205702 | 422 | 55.39 | 88.01 | 71.62 | 50.25 |
| LS6-LG3 | 420257 | 326764 | 421 | 55.14 | 91.75 | 78.84 | 77.75 |

HZ88-TG refers to PTMs living on the tubers of cultivar HZ-88, HZ88-LG refers to the midgut bacteria of PTMs living on the leaves of cultivar HZ-88. LS6-TG refers to midgut bacteria of PTMs living on the tubers of potato cultivar LS6, and LS6-LG refers to midgut bacteria of PTMs living on the leaves of potato cultivar LS6.
