# Supplementary material for: Midgut microbiota diversity of potato tuber moth associated with potato tissue consumed
Source: BMC Microbiol. 2020 Mar 11;20:58. doi: 10.1186/s12866-020-01740-8 (PMC7066784; doi:10.1186/s12866-020-01740-8)
Supplement: Supplementary file 6 — Additional file 6: Table S2. Number of analyzed 16S rRNA gene sequences of endophytic bacteria in leaves and tubers. [file 12866_2020_1740_MOESM6_ESM.docx]

**Additional file 6: Table S2.** Number of analyzed 16S rRNA gene sequences of endophytic bacteria in leaves and tubers

| Sample ID | PE Reads | Raw Tags | Clean Tags | AvgLen (bp) | GC (%) | Q20 (%) | Q30 (%) | Effective (%) |
| --- | --- | --- | --- | --- | --- | --- | --- | --- |
| HZ88-LG1 | 145051 | 137074 | 129926 | 440 | 53.51 | 96.37 | 92.96 | 87.27 |
| HZ88-LG2 | 80060 | 74942 | 69725 | 443 | 52.29 | 96.83 | 93.86 | 86.13 |
| HZ88-LG3 | 93434 | 89340 | 84775 | 444 | 52.48 | 96.93 | 94.09 | 89.07 |
| HZ88-TG1 | 269519 | 260052 | 244755 | 454 | 54.72 | 96.83 | 93.84 | 88.06 |
| HZ88-TG2 | 192471 | 186877 | 177535 | 452 | 54.13 | 96.9 | 94.00 | 88.88 |
| HZ88-TG3 | 167173 | 162000 | 153624 | 454 | 54.02 | 96.78 | 93.82 | 90.73 |
| LS6-LG1 | 139709 | 136417 | 132274 | 437 | 52.73 | 96.99 | 94.20 | 90.49 |
| LS6-LG2 | 112446 | 109237 | 105397 | 438 | 52.83 | 96.91 | 94.04 | 89.98 |
| LS6-LG3 | 121792 | 117348 | 112630 | 438 | 52.74 | 96.86 | 93.98 | 88.55 |
| LS6-TG1 | 224174 | 216859 | 203987 | 446 | 54.26 | 96.91 | 93.99 | 88.36 |
| LS6-TG2 | 187898 | 183416 | 176631 | 445 | 54.33 | 97.07 | 94.30 | 91.42 |
| LS6-TG3 | 203045 | 196264 | 185945 | 454 | 54.39 | 96.88 | 93.95 | 88.90 |

HZ88-LG refers to endophytic bacteria in the leaves of potato cultivar HZ-88, and HZ88-TG refers to endophytic bacteria in the tubers of potato cultivar HZ-88. LS6-LG refers to endophytic bacteria in the leaves of potato cultivar LS-6, and LS6-TG refers to endophytic bacteria in the tubers of potato cultivar LS-6.
