# Supplementary material for: Midgut microbiota diversity of potato tuber moth associated with potato tissue consumed
Source: BMC Microbiol. 2020 Mar 11;20:58. doi: 10.1186/s12866-020-01740-8 (PMC7066784; doi:10.1186/s12866-020-01740-8)
Supplement: Supplementary file 7 — Additional file 7: Table S3. The relative abundance of shared genera in different samples. [file 12866_2020_1740_MOESM7_ESM.docx]

**Additional file 7: Table S3.** The relative abundance of shared genera in different samples

|  | HZ88-LG (%) | HZ88-LE（%） | LS6-LG（%） | LS6-LE（%） | HZ88-TG  （%） | HZ88-TE （%） | LS6-TG（%） | LS6-TE（%） |
| --- | --- | --- | --- | --- | --- | --- | --- | --- |
| *Achromobacter* | 0.0939 | 0.2413 | 0.2007 | 0.1149 | 0.0849 | 0.0002 | 0.0721 |  |
| *Acinetobacter* | 2.2094 | 0.4400 | 1.6435 | 0.0342 | 5.8487 | 0.0002 | 12.1931 |  |
| *Alloprevotella* |  |  | 0.1078 | 0.0070 |  |  |  |  |
| *Aquabacterium* | 0.0338 | 0.0093 | 0.0848 | 0.0010 | 0.0092 |  |  |  |
| *Atopostipes* | 0.0261 | 0.0851 |  |  |  |  |  |  |
| *Bacillus* | 0.0083 | 0.1711 | 0.0983 | 0.4087 | 0.0047 | 0.0217 | 0.0022 | 0.0004 |
| *Bifidobacterium* |  |  | 0.1067 | 0.0130 |  |  |  |  |
| *Brachybacterium* | 0.0035 | 0.0131 | 0.0207 |  | 0.0060 |  | 0.0328 |  |
| *Bradyrhizobium* | 0.0067 | 0.1989 | 0.0188 | 0.0003 | 0.0056 | 0.0043 | 0.0039 | 0.0004 |
| *Brevundimonas* | 0.0260 | 0.1415 | 0.6108 | 4.8318 | 0.0244 | 0.0012 |  |  |
| *Cellvibrio* | 0.0175 | 0.0973 | 0.2958 | 0.0128 | 0.0023 |  | 0.1081 |  |
| *Chryseobacterium* | 0.1149 | 0.5363 | 1.1504 | 3.2136 | 0.1133 | 0.0003 | 0.0549 | 0.0006 |
| *Deinococcus* | 0.0260 | 0.0091 |  |  | 0.0013 |  |  |  |
| *Enhydrobacter* | 0.0261 | 0.0441 | 0.1411 | 0.0038 | 0.0100 | 0.0007 | 0.0006 |  |
| *Enterococcus* | 0.2144 | 0.1209 | 3.4692 | 0.0124 | 3.1538 | 5.9035 | 11.2466 | 1.1647 |
| *Escherichia_Shigella* | 78.2394 | 1.4557 | 55.8174 | 0.0186 | 0.1413 | 0.0021 | 0.0232 | 0.0036 |
| *Lactococcus* |  |  |  |  |  |  | 0.0399 | 0.0006 |
| *Flavobacterium* | 0.0001 | 0.1385 |  |  | 0.0604 |  | 0.2718 |  |
| *Halomonas* | 0.0291 | 0.0139 | 0.0441 | 0.0014 | 0.0021 | 0.0240 |  |  |
| *Lactobacillus* |  |  | 0.2281 | 0.0232 |  |  |  |  |
| *Lysobacter* |  |  | 0.0301 | 0.0339 | 0.0012 |  |  |  |
| *Methylobacterium* | 0.0632 | 0.1314 | 0.9917 | 0.2420 | 0.1288 | 0.0003 | 0.0660 |  |
| *Microbacterium* | 0.0008 | 0.0260 | 0.0597 | 1.0336 | 0.0013 | 0.0052 | 0.0004 | 0.0006 |
| *Moraxella* |  |  | 0.0208 | 0.1669 |  |  |  |  |
| *Neisseria* |  |  | 0.0227 |  | 0.0010 |  | 0.0004 | 0.0002 |
| *Nitrospira* |  |  | 0.0622 | 0.0058 |  |  |  |  |
| *Novosphingobium* | 0.0497 | 0.1716 | 0.1618 | 1.0726 | 0.0271 | 0.0002 | 0.0073 |  |
| *Ochrobactrum* | 0.2102 | 15.5229 | 1.0786 | 1.0010 | 0.0584 | 0.0393 | 0.1158 | 0.0103 |
| *Pantoea* | 0.0018 | 0.3072 | 0.0428 | 3.6308 | 0.0002 | 2.4477 |  |  |
| *Paracoccus* | 0.0153 | 0.1304 | 0.0146 | 0.1302 | 0.0107 | 0.0004 | 0.0828 |  |
| *Pectobacterium* | 0.0526 | 0.8322 |  |  |  |  | 2.3927 | 0.0004 |
| *Pedobacter* |  |  | 0.0787 | 1.0422 |  |  |  |  |
| *Planomicrobium* | 0.0170 | 0.0633 | 0.0069 | 0.0576 | 0.0021 | 0.0075 |  |  |
| *Propionibacterium* | 0.0001 | 0.2222 | 0.1078 | 0.0075 | 0.0002 | 0.0002 | 0.0006 |  |
| *Proteus* | 11.5028 | 0.0510 | 0.0094 | 0.0070 | 0.0635 |  | 0.0040 |  |
| *Pseudomonas* | 0.0985 | 1.3632 | 8.5730 | 6.6423 | 4.4717 | 17.0245 | 0.9381 | 5.2142 |
| *Ralstonia* | 0.1546 | 0.0144 | 0.2777 |  |  |  | 0.0207 |  |
| *Rhizobium* | 0.1649 | 5.7064 | 0.9280 | 41.8664 | 0.2864 | 0.0142 | 0.1675 | 0.0015 |
| *Rhodanobacter* | 0.0085% | 0.0265% | 0.0283 |  | 0.0007 |  | 0.0037 |  |
| *Rhodococcus* | 0.2379 | 0.0379 | 0.4005 | 0.0464 | 0.0009 | 0.0100 |  |  |
| *Serratia* | 0.0147 | 0.4634 | 0.0893 | 0.1247 | 0.0015 | 25.4419 |  |  |
| *Sphingobacterium* | 0.2223 | 0.1398 | 0.0724 | 4.9124 | 0.0488 | 0.0008 | 0.3460 |  |
| *Sphingobium* | 0.0017 | 0.0620 | 0.1108 | 0.2365 | 0.0152 |  |  |  |
| *Sphingomonas* | 1.9180 | 1.4712 | 2.2872 | 3.5500 | 0.1113 | 0.0009 | 0.3247 | 0.0002 |
| *Staphylococcus* | 0.0332 | 0.1995 | 0.1404 | 0.0351 | 0.0517 | 0.0327 | 0.0040 | 0.0203 |
| *Stenotrophomonas* | 0.0042 | 1.0981 | 0.5225 | 1.9686 | 0.0933 | 0.0057 | 0.1200 |  |
| *Streptococcus* |  |  | 0.0665 | 0.0166 |  |  |  |  |
| *Thermomonas* |  |  | 0.0507 | 0.0009 |  |  |  |  |
| *Tsukamurella* |  |  | 0.0585 | 0.0010 |  |  | 0.0052 | 0.0002 |
| *Variovorax* | 0.0950 | 0.0425 | 0.2558 | 0.0014 | 0.1340 |  | 0.0908 |  |
| *Weissella* | 0.0012 | 0.0198 | 0.1378 | 0.0066 |  |  | 0.0027 |  |

“-” indicates that this genus was not detected in this sample.

HZ88-TG refers to PTMs living on the tubers of cultivar HZ-88, HZ88-LG refers to the midgut bacteria of PTMs living on the leaves of cultivar HZ-88. LS6-TG refers to midgut bacteria of PTMs living on the tubers of potato cultivar LS6, and LS6-LG refers to midgut bacteria of PTMs living on the leaves of potato cultivar LS6. HZ88-LE refers to endophytic bacteria in the leaves of potato cultivar HZ-88, and HZ88-TE refers to endophytic bacteria in the tubers of potato cultivar HZ-88. LS6-LE refers to endophytic bacteria in the leaves of potato cultivar LS-6, and LS6-TE refers to endophytic bacteria in the tubers of potato cultivar LS-6.
